# Feeding ecology of a lesser‐known arboreal giant: Grizzled Giant Squirrel (*Ratufa macroura*), Southern Western Ghats, India

**DOI:** 10.1002/ece3.10765

**Published:** 2023-12-06

**Authors:** Kiran Thomas, Marek Šmejkal, Paingamadathil Ommer Nameer

**Affiliations:** ^1^ Institute of Hydrobiology Biology Centre of the Czech Academy of Sciences České Budějovice Czech Republic; ^2^ Faculty of Science University of South Bohemia České Budějovice Czech Republic; ^3^ Faculty of Forestry Kerala Agricultural University Thrissur India

**Keywords:** conservation, foraging behaviour, habitat use, riparian forest, Sciuridae‐Rodentia

## Abstract

Animal dietary choices help us understand a species' feeding behaviour and are particularly relevant in conservation management. The aim of this study was to gather knowledge on dietary choices and the foraging behaviour of the Grizzled Giant Squirrel (*Ratufa macroura*) in Chinnar Wildlife Sanctuary, Southern Western Ghats, Kerala, India. The objectives were in particular to determine the food composition, seasonal fluctuations in food selection and feeding technique. Through an observational sampling method, focal animal sampling, the Grizzled Giant Squirrel in Chinnar Wildlife Sanctuary was found to feed on 30 plant species belonging to 18 families. The most used plant family was Fabaceae, with eight species, followed by Moraceae (four species) and Anacardiaceae (two species). The food species consumed included 22 trees, four climbers, one liana, one paraphyte, one shrub and one succulent species. The squirrel spent the most time feeding on *Bauhinia racemosa* (19.79%), followed by *Tamarindus indica* (14.08%) and *Nothopegia beddomei* (9.89%). The squirrel's diet choice was primarily influenced by the availability of food tree species and food resources rather than the season and nontree species were also found in the diet of Grizzled Giant Squirrel. Although the Grizzled Giant Squirrel exhibits some plasticity in its dietary choices, the available diversity of mature trees and plants as food sources appears to be important for its conservation in the fragmented riparian forest of the Western Ghats in Southern India.

## Graphical abstract

This research enlightens the feeding ecology of the endangered Grizzled Giant Squirrel, which thrives in small riparian patches in the southern part of the Western Ghats, Kerala, India.

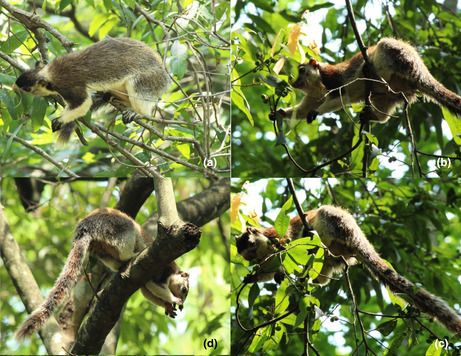

## 1 INTRODUCTION

Tree squirrels (order Rodentia) occur in almost every continent except Australia and Antarctica (Koprowski & Rajamani, 2008; Thorington et al., 2012) and provide critical ecosystem services, including seed dispersal and pollination (Hale & Koprowski, 2018; Miyaki, 1987; Steele et al., 2005; Zong et al., 2010). The giant squirrels are tree squirrels and the largest in the world, and they belong to the genus *Ratufa* (Thorington & Cifelli, 1989). The giant squirrels are ecologically, morphologically and zoo‐geographically unique, with their presence only in southeast Asia and serve as biological indicators of the habitat quality (Koprowski & Rajamani, 2008; Thorington & Cifelli, 1989).

Of the four giant arboreal squirrels belonging to the genus *Ratufa*, three are found in Indian landscapes, with Malabar Giant Squirrel (*Ratufa indica*) is endemic to the Indian subcontinent, the Malayan Giant Squirrel (*Ratufa bicolor*) is found in Northeast India and the Grizzled Giant Squirrel (*Ratufa macroura*) is endemic to Southern India and Sri Lanka (Menon, 2014). The Grizzled Giant Squirrel (hereafter GGS) is listed as a near‐threatened species (IUCN, 2022) and has three subspecies of which India harbours only one, *Ratufa macroura dandolena* (Ellerman, 1961; Johnsingh & Nameer, 2015; Menon, 2014). Mainly because of the sparse distribution of its habitat in its areas of occurrences, it shows one of the most remarkable examples of isolated populations among arboreal mammals (IUCN, 2019; Molur et al., 2005; Ramachandran, 1993). Due to habitat loss and hunting in most of its range, the remaining population in India are more threatened than in Sri Lanka (Goonatilake, 2019). Like most squirrels, it is primarily diurnal, but its highest activity has been observed in the early and late hours of the day (Paulraj, 1991).

In India, the Chinnar Wildlife Sanctuary in Kerala is home to the second largest population of GGS. The habitat of the animal is extremely unique and is confined primarily to a narrow stretch of riparian vegetation along the Chinnar and Pambar rivers and their major tributaries in the sanctuary (Ramachandran, 1993). Thus, the protection of these fragmented habitats is of prime importance for the conservation of the species. Habitat fragmentation, the main cause of the decline in arboreal giant squirrel populations, has become a hot subject among investigators and therefore studies on the population status of giant squirrels are extensive (Gurjar et al., 2013). In India, researchers contributed more to the population, distribution and ecology of *R. indica* compared with GGS (Ojha et al., 2023). Even though the GGS is the oldest recorded species of the genus *Ratufa*, dating back to 1769 (Ellerman et al., 1961), very little is known about its ecological aspects (Datta & Rajamani, 2015; Ojha et al., 2023). Very little published information is available on the feeding ecology of this species, including one study reporting 21 tree species forming the GGS diet (Kumar et al., 2007). An additional effort is warranted to achieve better understanding on its interaction with the ecosystem and preferred plant species (Kissling et al., 2014; Koprowski & Rajamani, 2008).

The ecological niche of an animal in an ecosystem can be revealed by understanding its diet and feeding behaviour (Bookhout, 1994; Dell'Agnello et al., 2019). Nutrient and energy contents play a major role in the selection of foods that the squirrels eat (Gurnell, 1987). Feeding of arboreal squirrels like GGS is confined to the middle canopy and very rarely the animal comes to the ground to feed on the scattered seeds or pursue other activities (Steele & Koprowski, 2003). Studies done in Srivilliputhur Grizzled Squirrel Sanctuary in Tamil Nadu, Southern India, showed that seeds and young fruits form the bulk of the GGS diet (Joshua, 1992). Based on a few literatures available on the feeding ecology of GGS, it is evident that it depends on available food resources in various sites of occurrence (Arya, 2018; Joshua, 1992; Kumar et al., 2007). Feeding techniques on a tree are related to the morphology and the mode of locomotion of the species (Clutton‐Brock & Harvey, 1977). Even though the feeding techniques of *R. indica* are described, there is a lack of literature evidence for the GGS (Ramachandran, 1992). The availability of food in an area is vital in supporting a minimum viable population for the squirrel to thrive (Palmer & Koprowski, 2014). Mammalian population dynamics are greatly dependent on and influenced by food availability and diet (Chapman et al., 2015). Therefore, the activity and foraging behaviour of squirrels may also get influenced by the differences in food availability and quality among habitats (Koli et al., 2013) Squirrels were found to concentrate their activities in regions with abundant food resources and avoid areas with inferior food sources (Lurz et al., 2000). Thus, the knowledge about the food preferences and feeding behaviour specific to the scattered locations where the GGS present is vital for its conservation and management in the country.

To protect and improve the conservation measures directed to an animal, especially a habitat specialist like the GGS, a proper understanding of the habitat requirement is vital (Mills, 1992). Here, we focussed on the foraging ecology of the GGS, to identify the food species and seasonal variation in food choice, diet composition and feeding technique. The diet and feeding behaviour of the GGS is not well known so far; thus, it would help in making specific management prescriptions for protecting the near‐threatened GGS in one of its prime refugia in India.

## 2 METHODS

### 2.1 Observation methods

**FIGURE 1:**
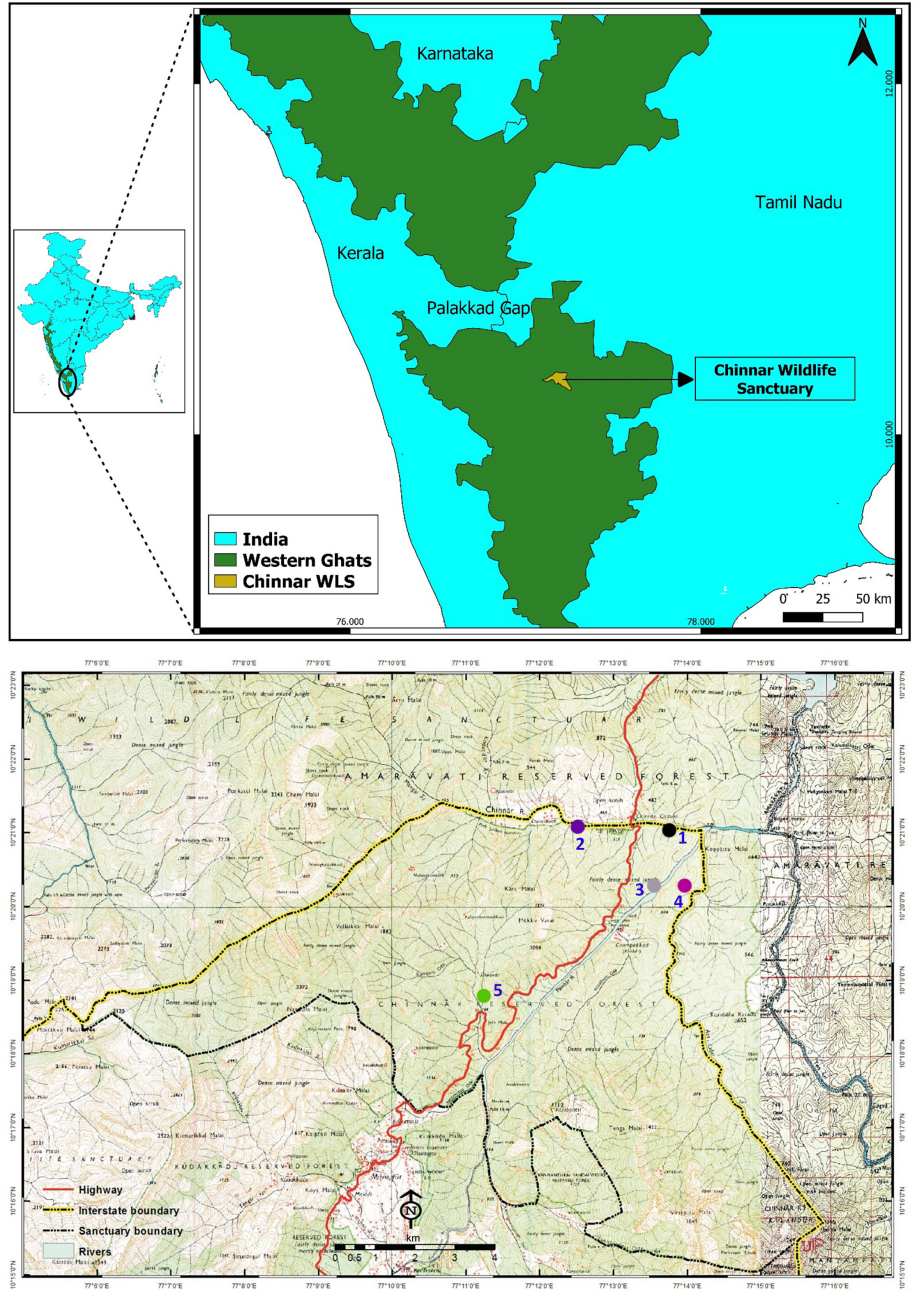
Study sites and the transect locations in the Chinnar Wildlife Sanctuary, southern Western Ghats, India.

The sites of the present study are in the riparian forests based on the knowledge of the occurrence of GGS in Chinnar Wildlife Sanctuary (Ramachandran, 1993; Figure 1). The information on the composition and seasonal variation in the GGS diet was collected through observational sampling. Focal animal sampling was used following Altmann (1974). Each encountered individual was followed, until it was out of sight and everything the animal did during the period was recorded. The individual‐level identification was not possible because of the type of vegetation and lack of expertise to easily distinguish the individuals only by direct observation. Two people were in the field, and only one (the same person) collected the data throughout the study period to avoid personal biases. Observation on the time spent on feeding, plant species eaten, plant part eaten, pickup rates of plant parts such as fruits, leaves, flowers, sap and bark were recorded. The data are structured as, encounters refer to how many times observer saw the squirrel (single individual or group entered as one encounter), and feeding incidences refers to number of feeding observations among all encounters. The feeding bouts mean the activities occur in bouts that are periods of, feeding activity within a food source or movement between two sources. The length of the bout was judged as the period between entry into and exit from a particular food source. During the period spent in the food source, some of the time was allotted to searching for a food item, selecting by smelling, remaining inactive or other grooming activities. The incidence of feeding on different food items across different seasons was categorised. This was done for three different seasons, summer (December–May), southwest monsoon (June–September) and northeast monsoon (October–November).

Observations were made for 10 months between April 2013 and May 2014, and within a month, at least 1 week was spent in the field. Five transects of 1000 m were laid after reconnaissance in the riparian habitat based on squirrel presence. Observations were made for each transect in both the forenoon and afternoon for an equal duration. Squirrels were observed using standard binoculars (model‐8x40 S, Olympus Global, Tokyo, Japan). The identification of the plant species was done through collected samples and by direct observation using plant identification database (Sasidharan, 1999, 2010) Different feeding postures and feeding techniques used by the GGS were interpreted by direct observation during the sampling.

### 2.2 Data analysis

To understand the diet composition of GGS, collected data were analysed by complementary approaches. The percentage contribution of different food items to the squirrel diet was calculated based on the duration of feeding on a particular item and the number of times of feeding incidence on a particular item. The Welch's *t*‐test was used to test any significant difference in feeding bout and duration, in forenoon and afternoon hours. A circular analysis, Rayleigh test was done to check the association of a plant species fed by the GGS and seasons. A distance‐based RDA analysis (similarity coefficient—Bray–Curtis) was done to depict the species usage by GGS against the seasons and time of the day as factors using Canoco5 (Braak & Smilauer, 2012; Legendre & Anderson, 1999).

## 3 RESULTS

### 3.1 Food composition

The GGS was found to be feeding on different plant parts, including the leaves, seeds, flowers, sap and bark of different trees and other vegetation (Table 1). A total of 1314 min (in 10 months) in 42 encounters of feeding observation were collected during the study. A total of 30 different species of vegetation, including 22 tree species, four climbers, one each of liana, paraphyte, shrub and cactus, formed the diet. The maximum duration of feeding was observed on *Bauhinia racemosa* (19.79%), followed by *Tamarindus indica* (14.08%), *Nothopegia beddomei* (9.89%), *Strychnos potatorum* (7.23%) and *Terminalia arjuna* (6.47%; Table 1).

**TABLE 1:** Details of each feeding incident of the Grizzled Giant Squirrel on a particular species.

Species	Family	Month	Part eaten	Total duration (min)	Duration of feeding (%)
*Mangifera indica*	Anacardiaceae	October	Leaves and flowers	15	1.14
*Nothopegia beddomei*	Anacardiaceae	May	Immature seeds	130	9.89
*Commiphora caudata*	Burseraceae	March	Bark and tender leaves	10	0.76
*Terminalia arjuna*	Combretaceae	April, September, January	Leaves and fruits	85	6.47
*Diplocyclos palmatus*	Cucurbitaceae	November	Immature leaves and seeds	45	3.42
*Hopea parviflora*	Dipterocarpaceae	February	Leaves	8	0.61
*Euphorbia trigona*	Euphorbiaceae	August	Leaves	27	2.05
*Acacia* spp.	Fabaceae	March	Seeds of immature fruit	35	2.66
*Albizia lebbeck*	Fabaceae	March	Tender leaves and flowers	36	2.74
*Bauhinia racemosa*	Fabaceae	February, March	Immature seeds	260	19.79
*Derris brevipes*	Fabaceae	March	Tender leaves and flowers	17	1.29
*Entada rheedii*	Fabaceae	March	Tender leaves and flowers	15	1.14
*Pongamia pinnata*	Fabaceae	April	Immature leaves	23	1.75
*Tamarindus indica*	Fabaceae	November, December, May	Seeds of immature fruit and flowers	185	14.08
*Cassia fistula*	Fabaceae	May	Bark	3	0.23
*Strychnos potatorum*	Loganiaceae	January	Immature seed	95	7.23
*Macrosolon capitellatus*	Loranthaceae	May	Immature seeds	55	4.19
*Hibiscus rosa*—*sinensis*	Malvaceae	March	Tender leaves and flowers	15	1.14
*Melia dubia*	Meliaceae	September	Leaves	37	2.82
*Ficus albiphyla*	Moraceae	February	Tender leaves and bark	40	3.04
*Ficus microcarpa*	Moraceae	November, February	Leaves and immature seed	29	2.21
*Ficus* spp.1	Moraceae	November	Leaves and immature seed	16	1.22
*Ficus* spp.2	Moraceae	September	Leaves	16	1.22
*Syzygium cumini*	Myrtaceae	August, May	Seeds of immature and mature fruits and leaves	52	3.96
*Psychotria subintegra*	Rubiaceae	September	Leaves, flowers and sap	13	0.99
*Aegle marmelos*	Rutaceae	October	Leaves	3	0.23
*Santalum album*	Santalaceae	November	Immature leaves and flowers	9	0.68
*Grewia tiliifolia*	Tiliaceae	October	Leaves, flowers and fruits	25	1.90
*Grewia* spp.	Tiliaceae	May	Leaves	5	0.38
*Cayratia trifolia*	Vitaceae	March	Tender leaves and flowers	10	0.76

Feeding varied according to the availability of food in the different months. The GGS fed on *Terminalia arjuna* (January, April and September) and *Tamarindus indica* (May, November and December) in 3 different months, followed by *Bauhinia racemosa* (February and March), *Ficus microcarpa* (February and November) and *Syzygium cumini* (August, May) in 2 different months. However, the other species were observed to be eaten only once during the study period. Climbing plants that were part of the diet included *Derris brevipes*, *Diplocyclos palmatus* and *Cayratia trifolia*. The GGS also used shrubs such as *Hibiscus rosa*—*sinensis*, cacti—*Euphorbia trigona* and liana—*Entada rheedii* as food. These species belong to 18 different families. Among them, the preferred family was Fabaceae, with eight species, followed by Moraceae, with four species, and Anacardiaceae, with two species.

### 3.2 Contribution of different vegetation parts

**FIGURE 2:**
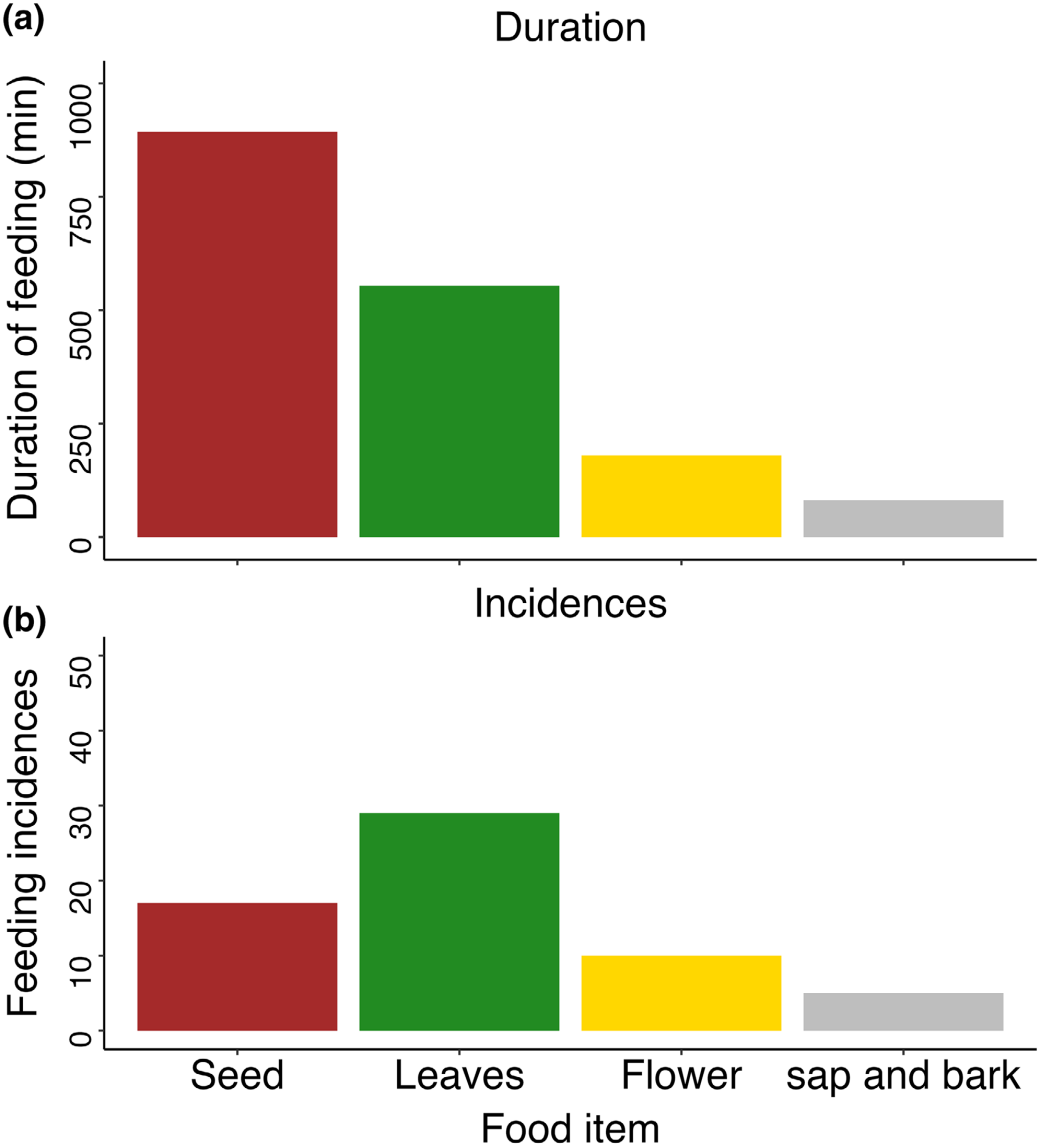
Contribution of each food item to total feeding based on (a) the duration of feeding on a particular item and (b) the number of times feeding on a particular item occurred.

The total number of feeding incidences by type of plant part consumed, during the entire study period, was 62, of which 30 incidences were leaf feeding (48.39%), followed by seed feeding (27.32%), flower feeding (16.13%), and sap and bark‐feeding (8.06%; Figure 2a). The duration of feeding on different objects is seeds (52.12%), followed by leaves (32.55%), flowers (10.58%) and sap and bark (4.76%; Figure 2b).

### 3.3 Diurnal variation in feeding habit

The feeding duration recorded for the entire study period was 1314 min, including 915 feeding bouts. From the total feeding duration, 42% was recorded in the forenoon and the remaining 58% in the afternoon, with the sampling hours being the same for both times of day. During the morning feedings, 321 feeding bouts were counted, while in the afternoon approximately double was recorded with 594 bouts. However, the GGS were found to be inactive at a food source for some time before the start of the feeding or after feeding, before moving on to search for a new source or for other activities. In some cases, the GGS were found to be inactive or resting at a food source between periods of intensive feeding. The duration of feeding (*t* = 1.4649, *p* = .1524) and the feeding bout (*t* = 1.1923, *p* = .2424) between the forenoon and afternoon hours were found nonsignificant.

### 3.4 Seasonal variation in diet

**FIGURE 3:**
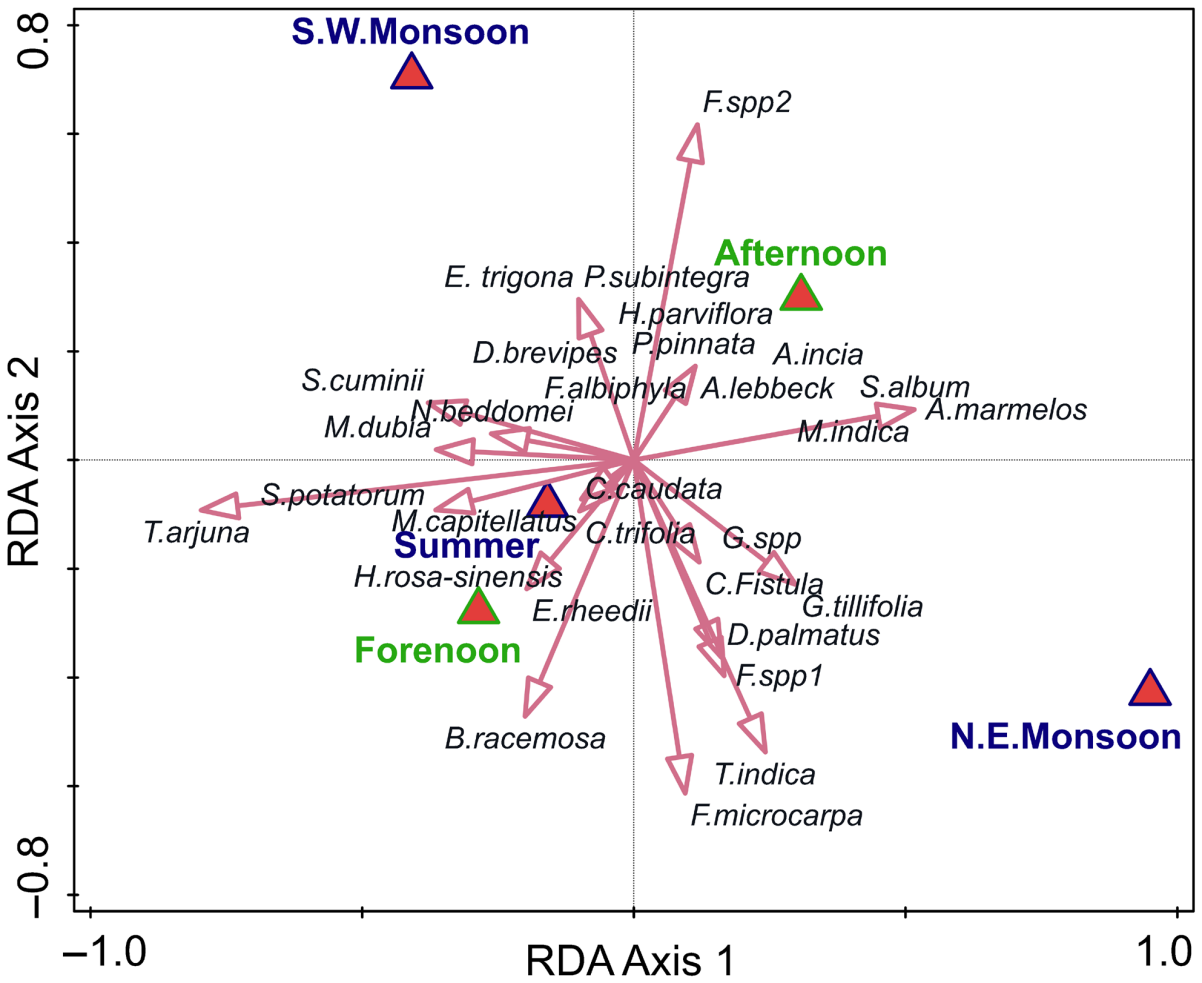
Distance‐based redundancy analysis shows the association of season and time of day with the plant species fed by the Grizzled Giant Squirrel. The presence of the plant species in the diet was analysed against the seasons as one factor, northeast monsoon, southwest monsoon and summer (three levels) and time of the day as another factor, forenoon and afternoon (two levels). The distance matrix used was Bray–Curtis.

The frequency of feeding on all foods was highest in summer, followed by the northeast and southwest monsoons. Circular analysis showed that there was no concentration of data around a particular month of observation (*p* = .0843). Distance‐based RDA revealed that the selection of plant species does not change systematically between seasons (and within a day). The GGS either choose the plant species at random or their choice is influenced by other aspects (e.g. when the fruits are ripe; Figure 3).

### 3.5 Feeding technique

**FIGURE 4:**
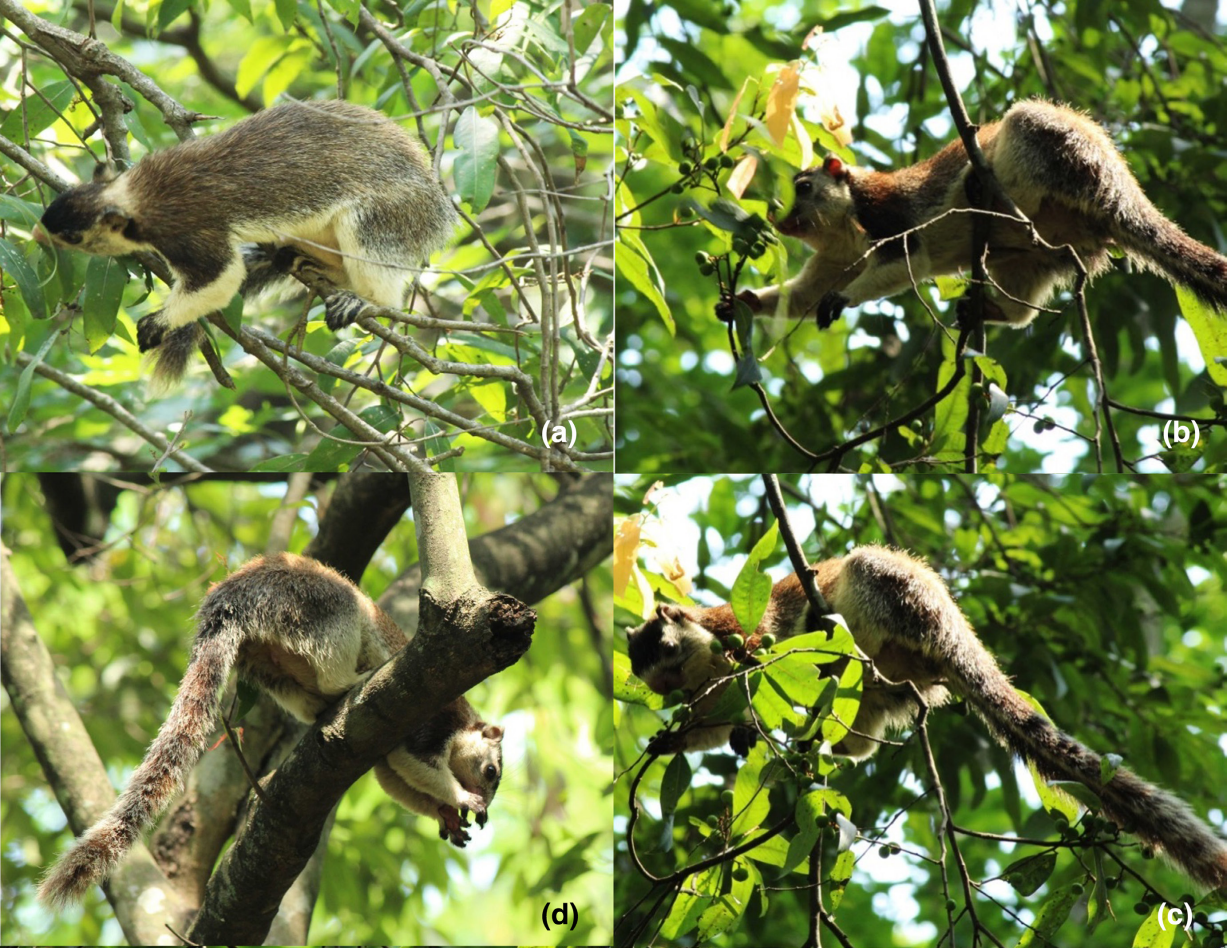
Feeding techniques: (a) selection by smell, (b) making the food reach to mouth with the help of forelimbs, (c) cutting the fruit with the mouth and (d) feeding the seeds by sitting on a firm branch.

The GGS are very selective in their choice of food. Food selection seems to be based on odour (Figure 4a). The GGS were found to be handling its food with both mouth and forelimbs (Figure 4b). Depending on the availability of food sources, the GGS moves to the top of the branch or other areas of the tree canopy and cuts the pod, fruit, leaves or flower with its mouth (Figure 4c). Occasionally, the food is brought to the mouth with the help of the forelimbs. With the fruit in its mouth, the squirrel then moves to the thick horizontal branch to latch on, sometimes feeding began at the point of harvest. The squirrel holds the branch with the claws of the hind limbs and the hanging tail, which gives the body further balance (Figure 4d). It then gnaws on the epicarp of the fruit to extract and consume the seed. During feeding, the forelimbs were effectively used to manipulate the food, be it long pods of *Bauhinia racemosa* or small fruits of *Grewia tiliifolia*.

### 3.6 Feeding posture

Three main postures were observed in the present study. The most common feeding posture is that of the squirrel sitting on a horizontal branch of the tree and eating and the second posture is that of the animal hanging upside down on small branches, supporting itself with its hind limbs and tail, reaching for the food in the hanging posture. In the third feeding posture, the squirrel holds on the bole of the tree and eats upside down.

**FIGURE 5:**
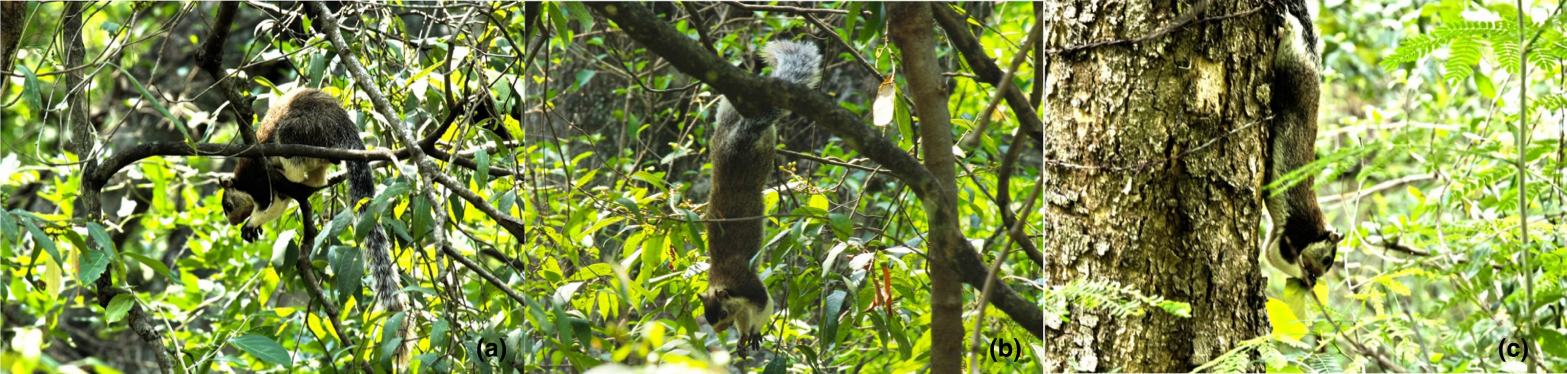
Feeding postures: (a) sitting posture, (b) hanging posture and (c) clinging posture.

In most observations of feeding posture, it was found that squirrels eat sitting on the branch, holding the branch with their hind legs and their tail hanging down. The hanging tail helps the animal to balance its body. This type of posture was observed in feeding *Bauhinia racemosa* and *Grewia tiliifolia* (Figure 5a). The second posture consisted of GGS feeding hanging upside down from the branch using the claws of the hind legs, while the tail remained curled over the branch. This posture was mainly observed when feeding on fruit and flower clusters on small branches that could not support the squirrel's body weight. Sometimes this posture was also adopted just for harvesting. This was observed when GGS fed on *Strychnos potatorum* and *Nothopegia beddomei* (Figure 5b).

The third feeding posture observed was a GGS lying on the vertical bole, holding on to the bole with the claws of its hind legs and manipulating the food with its mouth and forelegs (Figure 5c). In the observed cases, the animals fed on the climbing plants they found on the trees or the tree sap and bark.

## 4 DISCUSSION

In the present study, GGS were found to feed on 30 different vegetation species including 22 tree species and eight species including climbers, lianas, paraphytes, shrubs and cacti in Chinnar Wildlife Sanctuary, Western Ghats, South India. Compared with an earlier report, in which 21 tree species were mentioned (Kumar et al., 2007), eight more species have been added. The GGS feed mainly on the seeds, tender leaves, flowers, bark and sap of these species, whereas in previous studies there was no mention of the GGS eating the plants or plant parts. The squirrels were observed to eat on tender leaves and flowers of plant species such as *Derris brevipes* and *Diplocyclos palmatus* (climbers), *Entada rheedii* (liana), *Hibiscus rosa*—*sinensis* (shrub) and *Euphorbia trigona* (cactus). In comparison with Joshua (1992) who reported that GGS fed on the tender leaves and flowers of two trees, *Tamarindus indica* and *Bauhinia purpurea*, our results showed that GGS fed extensively on the tender leaves of most of the tree species, which contributed to its diet.

### 4.1 Food composition

The theory of optimal foraging proposed by Pyke (1984) states that a forager should only eat the most preferred or highest ranked item if a sufficient amount of that item is available to meet its daily nutritional requirements. When the preferred food item is exhausted, the forager should include the next highest‐ranking item in its diet. The percentage of different plant parts suggests that the GGS prefer more seeds in their diet and may switch more to leafy foods, flowers, sap and bark when seeds are scarce or unavailable. However, the present observation confirms the findings of (Thorington & Cifelli, 1989) on *R. indica* in Mudumalai Wildlife Sanctuary and (Ramachandran, 1992) in Periyar, Parambikulam and Silent Valley. Thus, in the present study, we found that the GGS acts as an important seed disperser for many of the tree/plant species in the riverine vegetation.

Rodents rely heavily on hoarding for their survival and reproduction (Vander Wall, 1990; Wang et al., 2018). In contrast to a report of hoarding by *R. indica* (Somanathan et al., 2007), there was no case of hoarding by GGS during the study period. This could either be a clear indication of optimal food availability in the habitat (Ando et al., 1985) or that GGS does not have this habit. Further studies on the energy budget of GGS are required, as this animal appears to be a plant generalist in this study area, feeding on seeds, leaves, flowers, sap and bark.

### 4.2 Diurnal variation in feeding habit

The feeding observations over different hours of the day show that the animal was very active in the morning and evening hours of the day and rested during midday. The feeding activity thus seemed to be influenced by the weather factors in the region. In the hours when the sun was shining strongly or it was raining, the activity of the GGS was very low. Temperature‐dependent activity patterns to avoid extreme weather conditions have already been observed in diurnal squirrels (Lee et al., 1990; Skibiel et al., 2002).

### 4.3 Feeding posture and technique

The mouth and the forelimbs are the organs that help in handling the food, while the hind limbs and the tail serve as supporting organs for the balance of the body during feeding. It has been found that all three feeding postures, such as the sitting, hanging and clinging postures, are effectively utilised by the animal. This depends on the availability and type of food found. Similar feeding postures were reported by (Ramachandran, 1992) on *Ratufa R. indica*. Besides being the second largest GGS population in the country, Chinnar Wildlife Sanctuary has a very small piece of riparian habitat (1.6 km^2^) that harbours a very small GGS population and is already under serious threat of habitat fragmentation and degradation (Thomas & Nameer, 2018). Further habitat fragmentation and degradation will lead to increased food scarcity and predation risk (Gurjar et al., 2013; Joshua & Johnsingh, 1994; Thomas et al., 2017). Food scarcity causes animals to increase the duration of food intake, which is positively correlated with stress (Dunn et al., 2010; Laurance et al., 2011). Habitat fragmentation accelerates the death of large and mature trees (Laurance et al., 2000), which has also been observed in the Chinnar Wildlife Sanctuary (Thomas & Nameer, 2021). These trees contain a greater amount of food resources (Chapman et al., 1992) and can potentially limit the availability of preferred food for GGS. Aside from food sources, GGS are associated with many of the riparian tree species for their drey construction and survival (Thomas & Nameer, 2021). Considering these factors, up‐to‐date information on the vegetation types with which GGS interact is essential for their conservation management.

## 5 CONCLUSIONS AND RECOMMENDATIONS

This study adds to the knowledge of GGS diet by expanding the number of known food types and emphasising the importance of food availability over seasonality, in a given habitat. This attempt is the first to detail the feeding technique and feeding postures of this near‐threatened squirrel at this study site. It also highlights the potential food shortage that this species may face in the near future if habitat degradation cannot be curbed in Chinnar Wildlife Sanctuary, Kerala, India. A detailed study of the energetics of GGS along with site‐specific habitat restoration, including the species associated with the ecology of GGS in this particular habitat, can ensure the long‐term survival of this squirrel.

## AUTHOR CONTRIBUTIONS

**Kiran Thomas:** Conceptualization (equal); data curation (lead); formal analysis (lead); investigation (lead); visualization (lead); writing – original draft (lead). **Marek Šmejkal:** Validation (equal); visualization (equal); writing – review and editing (lead). **Paingamadathil Ommer Nameer:** Conceptualization (equal); funding acquisition (lead); methodology (lead); writing – review and editing (supporting).

## CONFLICT OF INTEREST STATEMENT

The authors declare no conflicts of interest.

## Data Availability

The data supporting this study's findings are openly available in Dryad at https://doi.org/10.5061/dryad.gtht76hsq.

## References

[ece310765-bib-0001] Altmann, J. (1974). Observational study of behavior: Sampling methods. Behaviour, 49(3–4), 227–266. 10.1163/156853974X00534 4597405

[ece310765-bib-0002] Ando, M. , Shiraishi, S. , & Uchida, T. A. (1985). Feeding behaviour of three species of squirrels. Behaviour, 95(1–2), 76–86. 10.1163/156853985X00055

[ece310765-bib-0003] Arya, U. (2018). Ecology of grizzled giant squirrel in Cauvery Wildlife Sanctuary, Karnataka, Southern India . Indian Institute of Science Education and Research (IISER) Pune.

[ece310765-bib-0004] Bookhout, T. A. (1994). Research and management techniques for wildlife and habitats. Wildlife Society.

[ece310765-bib-0005] Braak, C. J. F. , & Smilauer, P. (2012). Canoco reference manual and user's guide: Software for ordination (version 5.0). Biometris.

[ece310765-bib-0006] Chapman, C. A. , Chapman, L. J. , Wangham, R. , Hunt, K. , Gebo, D. , & Gardner, L. (1992). Estimators of fruit abundance of tropical trees. Biotropica, 24, 527–531.

[ece310765-bib-0007] Chapman, C. A. , Schoof, V. A. M. , Bonnell, T. R. , Gogarten, J. F. , & Calmé, S. (2015). Competing pressures on populations: Long‐term dynamics of food availability, food quality, disease, stress and animal abundance. Philosophical Transactions of the Royal Society, B: Biological Sciences, 370(1669), 20140112. 10.1098/rstb.2014.0112 PMC4410378 25870398

[ece310765-bib-0008] Clutton‐Brock, T. H. , & Harvey, P. H. (1977). Species differences in feeding and ranging behavior in primates. In T. H. Clutton‐Brock (Ed.), Primate ecology (pp. 557–584). Academic Press.

[ece310765-bib-0009] Datta, A. , & Rajamani, N. (2015). Sciurids (pp. 513–573). Universities Press.

[ece310765-bib-0010] Dell'Agnello, F. , Natali, C. , Bertolino, S. , Fattorini, L. , Fedele, E. , Foggi, B. , Martini, M. , Pisani, C. , Riga, F. , Sgarlata, A. , Ciofi, C. , & Zaccaroni, M. (2019). Assessment of seasonal variation of diet composition in rodents using DNA barcoding and Real‐Time PCR. Scientific Reports, 9(1), 14124. 10.1038/s41598-019-50676-1 31575934 PMC6773709

[ece310765-bib-0011] Dunn, J. C. , Cristóbal‐Azkarate, J. , & Veà, J. J. (2010). Seasonal variations in the diet and feeding effort of two groups of howlers in different sized forest fragments. International Journal of Primatology, 31(5), 887–903. 10.1007/s10764-010-9436-0

[ece310765-bib-0012] Ellerman, J. R. (1961). Review of “the Fauna of India including Pakistan, Burma and Ceylon: Mammalia (second edition). Vol. 3. Rodentia”. The Journal of the Bombay Natural History Society, 61, 676–680.

[ece310765-bib-0013] Ellerman, J. R. , Roonwal, M. L. , Biswas, B. , Blanford, W. T. , & Zoological Survey of India . (1961). The fauna of India, including Pakistan, Burma and Ceylon: Mammalia. Rodentia. Vol. 3. Zoological Survey of India.

[ece310765-bib-0014] Goonatilake, S. D. A. (2019). Ratufa macroura . The IUCN Red List of Threatened Species 2019. 10.2305/IUCN.UK.2019-1.RLTS.T19381A22261644.en

[ece310765-bib-0015] Gurjar, R. L. , Kumbhar, A. S. , Jena, J. , Yogesh, J. K. , Dave, C. , Singh, R. P. , & Mitra, A. (2013). Population density of Indian giant squirrel *Ratufa indica centralis* (Ryley, 1913) in Satpura National Park, Madhya Pradesh, India. Journal of Research in Biology, 3, 1086–1092.

[ece310765-bib-0016] Gurnell, J. (1987). The natural history of squirrels. Christopher Helm Ltd.

[ece310765-bib-0017] Hale, S. L. , & Koprowski, J. L. (2018). Ecosystem‐level effects of keystone species reintroduction: A literature review. Restoration Ecology, 26(3), 439–445. 10.1111/rec.12684

[ece310765-bib-0018] IUCN . (2019). IUCN SSC Small Mammal Specialist Group. Ratufa macroura . The IUCN Red List of Threatened Species. Version 2022‐2.

[ece310765-bib-0019] IUCN . (2022). The IUCN Red List of Threatened Species . Version 2022‐2. https://www.iucnredlist.org

[ece310765-bib-0020] Johnsingh, A. J. T. , & Nameer, P. O. (2015). Grizzled giant squirrel. In A. J. T. Johnsingh & N. Manjrekar (Eds.), Mammals of South Asia (p. 799). University Press.

[ece310765-bib-0021] Joshua, J. (1992). Ecology of the endangered grizzled giant squirrel (Ratufa macroura) in Tamil Nadu, South India .

[ece310765-bib-0022] Joshua, J. , & Johnsingh, A. J. T. (1994). Impact of biotic disturbances on the habitat and population of the endangered grizzled giant squirrel *Ratufa macroura* in South India. Biological Conservation, 68(1), 29–34. 10.1016/0006-3207(94)90543-6

[ece310765-bib-0023] Kissling, W. D. , Dalby, L. , Fløjgaard, C. , Lenoir, J. , Sandel, B. , Sandom, C. , Trøjelsgaard, K. , & Svenning, J. (2014). Establishing macroecological trait datasets: Digitalization, extrapolation, and validation of diet preferences in terrestrial mammals worldwide. Ecology and Evolution, 4(14), 2913–2930. 10.1002/ece3.1136 25165528 PMC4130448

[ece310765-bib-0024] Koli, V. K. , Bhatnagar, C. , & Sharma, S. K. (2013). Food habits of Indian giant flying squirrel (*Petaurista philippensis* Elliot) in tropical deciduous forest, Rajasthan, India. Mammal Study, 38(4), 251–259. 10.3106/041.038.0409

[ece310765-bib-0025] Koprowski, J. , & Rajamani, N. (2008). Global hotspots and knowledge gaps for tree and flying squirrels. Current Science, 95, 851–856.

[ece310765-bib-0026] Kumar, S. , Agoramoorthy, G. , & Hsu, M. J. (2007). Population size, density and conservation status of the grizzled giant squirrel in Chinnar Wildlife Sanctuary, India. Mammalia, 71(1/2), 89–94. 10.1515/MAMM.2007.009

[ece310765-bib-0027] Laurance, W. F. , Camargo, J. L. C. , Luizão, R. C. C. , Laurance, S. G. , Pimm, S. L. , Bruna, E. M. , Stouffer, P. C. , Bruce Williamson, G. , Benítez‐Malvido, J. , Vasconcelos, H. L. , Van Houtan, K. S. , Zartman, C. E. , Boyle, S. A. , Didham, R. K. , Andrade, A. , & Lovejoy, T. E. (2011). The fate of Amazonian forest fragments: A 32‐year investigation. Biological Conservation, 144(1), 56–67. 10.1016/j.biocon.2010.09.021

[ece310765-bib-0028] Laurance, W. F. , Delamônica, P. , Laurance, S. G. , Vasconcelos, H. L. , & Lovejoy, T. E. (2000). Rainforest fragmentation kills big trees. Nature, 404(6780), 836. 10.1038/35009032 10786782

[ece310765-bib-0029] Lee, T. M. , Homes, W. G. , & Zucker, I. (1990). Temperature dependence of circadian rhythms in golden‐mantled ground squirrels. Journal of Biological Rhythms, 5(1), 25–34. 2133117 10.1177/074873049000500103

[ece310765-bib-0030] Legendre, P. , & Anderson, M. J. (1999). Distance‐based redundancy analysis: Testing multispecies responses in multifactorial ecological experiments. Ecological Monographs, 69(1), 1. 10.2307/2657192

[ece310765-bib-0031] Lurz, P. W. W. , Garson, P. J. , & Wauters, L. A. (2000). Effects of temporal and spatial variations in food supply on the space and habitat use of red squirrels (*Sciurus vulgaris* L.). Journal of Zoology, 251(2), 167–178.

[ece310765-bib-0032] Menon, V. (2014). A field guide to Indian mammals. Darling Kindersley (India) Pvt. Ltd. and Penguin Book of India (P.) Ltd.

[ece310765-bib-0033] Mills, M. G. L. (1992). A comparison of methods used to study food habits of large African carnivores. In D. R. McCullough & R. H. Barrett (Eds.), Wildlife 2001: Populations (pp. 1112–1124). Springer Netherlands. 10.1007/978-94-011-2868-1_85

[ece310765-bib-0034] Miyaki, M. (1987). Seed dispersal of the Korean pine, *Pinus koraiensis*, by the red squirrel, *Sciurus vulgaris* . Ecological Research, 2(2), 147–157. 10.1007/BF02346923

[ece310765-bib-0035] Molur, S. , Srinivasulu, C. , Srinivasulu, B. , Walker, S. , Nameer, P. O. , & Ravikumar, L. (2005). Status of non‐volant small mammals: Conservation Assessment and Management Plan (C.A.M.P) workshop report .

[ece310765-bib-0036] Ojha, S. P. , Chetia, H. , Sarma, K. , & Chatakonda, M. K. (2023). Sciuridae research in South Asia—A short review. Proceedings of the Zoological Society, 76, 207–215. 10.1007/s12595-023-00481-6

[ece310765-bib-0037] Palmer, R. R. , & Koprowski, J. L. (2014). Feeding behavior and activity patterns of Amazon red squirrels. Mammalia, 78(3), 303–313. 10.1515/mammalia-2013-0083

[ece310765-bib-0038] Paulraj, S. (1991). Grizzled giant squirrel in the final throes of the extinction process. Zoos' Print, 6, 1–2.

[ece310765-bib-0039] Pyke, G. H. (1984). Optimal foraging theory: A critical review. Annual Review of Ecology and Systematics, 15(1), 523–575. 10.1146/annurev.es.15.110184.002515

[ece310765-bib-0040] Ramachandran, K. K. (1992). Certain aspects of ecology and behaviour of Malabar giant squirrel, Ratufa indica (Schreber) . University of Kerala.

[ece310765-bib-0041] Ramachandran, K. K. (1993). Status survey and distribution of endangered grizzled giant squirrel in Chinnar Wildlife Sanctuary, Kerala, India. Indian Journal of Forestry, 16(3), 226–231.

[ece310765-bib-0042] Sasidharan, N. (1999). Study on the flora of Chinnar Wildlife Sanctuary . PMC3331175 22556803

[ece310765-bib-0043] Sasidharan, N. (2010). Forest trees of Kerala (2nd ed.). Kerala Forest Research Institute.

[ece310765-bib-0044] Skibiel, A. , Superka, B. , & Swan, C. (2002). Is squirrel foraging activity associated with temperature. Journal of Ecological Research, 4, 5–8.

[ece310765-bib-0045] Somanathan, H. , Mali, S. , & Borges, R. M. (2007). Arboreal larder‐hoarding in the tropical Indian giant squirrel *Ratufa indica* . Ecoscience, 14(2), 165–169.

[ece310765-bib-0046] Steele, M. , Wauters, L. A. , & Larsen, K. W. (2005). Selection, predation and dispersal of seeds by tree squirrels in temperate and boreal forests: Are tree squirrels keystone granivores? In P. M. Forget , J. Lambert , P. Hulme , & S. Vander Wall (Eds.), Seed fate: Predation, dispersal and seedling establishment (pp. 205–221). CABI Publishing. 10.1079/9780851998060.0205

[ece310765-bib-0047] Steele, M. A. , & Koprowski, J. L. (2003). North American tree squirrels. Smithsonian Books.

[ece310765-bib-0048] Thomas, K. , Das, A. A. , & Nameer, P. O. (2017). A report on the predation of grizzled giant squirrel (*Ratufa macroura*) by changeable hawk‐eagle (*Nisaetus cirrhatus*), from Western Ghats, South India. Zoos' Print, 32(4), 11–14.

[ece310765-bib-0049] Thomas, K. , & Nameer, P. O. (2018). Alarming population status of the grizzled giant squirrel *Ratufa macroura* (Mammalia: Rodentia: Sciuridae) in Chinnar Wildlife Sanctuary, the Western Ghats, India. Journal of Threatened Taxa, 10(10), 12350–12356. 10.11609/jott.3536.10.10.12350-12356

[ece310765-bib-0050] Thomas, K. , & Nameer, P. O. (2021). Characterisation of breeding habitat of grizzled giant squirrel *Ratufa macroura* (Mammalia: Sciuridae) in Chinnar Wildlife Sanctuary, Western Ghats, India. Journal of Threatened Taxa, 13(8), 18993–19001. 10.11609/jott.7371.13.8.18993-19001

[ece310765-bib-0051] Thorington, R. W., Jr. , Koprowski, J. L. , Steele, M. A. , & Whatton, J. F. (2012). Squirrels of the world. JHU Press.

[ece310765-bib-0052] Thorington, R. W., Jr. , & Cifelli, R. L. (1989). The usual significance of the giant squirrels (*Ratufa*). In J. C. Daniel & J. S. Serrao (Eds.), Conservation in developing countries: Problem and prospects. Proceeding of the Centenary Seminar of the Bombay Natural History Society (pp. 212–219). Oxford University Press.

[ece310765-bib-0053] Vander Wall, S. B. (1990). Food hoarding in animals. University of Chicago Press.

[ece310765-bib-0054] Wang, Z. , Wang, B. , Yi, X. , Yan, C. , Cao, L. , & Zhang, Z. (2018). Scatter‐hoarding rodents are better pilferers than larder‐hoarders. Animal Behaviour, 141, 151–159. 10.1016/j.anbehav.2018.05.017

[ece310765-bib-0055] Zong, C. , Wauters, L. A. , Van Dongen, S. , Mari, V. , Romeo, C. , Martinoli, A. , Preatoni, D. , & Tosi, G. (2010). Annual variation in predation and dispersal of Arolla pine (*Pinus cembra* L.) seeds by Eurasian red squirrels and other seed‐eaters. Forest Ecology and Management, 260(5), 587–594. 10.1016/j.foreco.2010.05.014

